# Differences Between Omnivores and Vegetarians in Personality Profiles, Values, and Empathy: A Systematic Review

**DOI:** 10.3389/fpsyg.2021.579700

**Published:** 2021-10-07

**Authors:** Sophie Holler, Holger Cramer, Daniela Liebscher, Michael Jeitler, Dania Schumann, Vijayendra Murthy, Andreas Michalsen, Christian S. Kessler

**Affiliations:** ^1^Institute of Social Medicine, Epidemiology and Health Economics, Charité – Universitätsmedizin Berlin, Corporate Member of Freie Universität Berlin and Humboldt-Universität zu Berlin, Berlin, Germany; ^2^Department of Internal and Integrative Medicine, Evang. Kliniken Essen-Mitte, Faculty of Medicine, University of Duisburg-Essen, Essen, Germany; ^3^Australian Research Centre in Complementary and Integrative Medicine, University of Technology Sydney, Sydney, NSW, Australia; ^4^National Centre for Naturopathic Medicine, Southern Cross University, Lismore, NSW, Australia; ^5^Department of Internal and Complementary Medicine, Immanuel Hospital Berlin, Berlin, Germany

**Keywords:** vegetarian, plant-based, omnivore, diet, personality, values, empathy

## Abstract

Numerous medical studies have documented vegetarian diets as having various health benefits. Studies have also compared vegetarians with other dietary groups from a socio-psychological perspective. The objective of this review is to investigate the differences between vegetarians and omnivores in terms of their personality profiles, values, and empathy skills. A search was conducted across three electronic databases. Non-randomized, observational, cross-sectional, and cohort studies were eligible. Outcomes provided information about the differences between the above-mentioned dietary groups regarding their personality profiles, values, and empathy skills. A shortened version of the Newcastle–Ottawa Scale was used to assess the risk of bias for the included studies. Of the 2,513 different studies found, 25 (total number of participants *n* = 23,589) were ultimately included. These studies indicate that vegetarians significantly differ from omnivores in their personalities, values, and ability to be empathetic. Omnivorism is associated with an increased orientation toward social dominance, greater right-wing authoritarianism, and, in line with this, a stronger tendency to be prejudiced. Vegetarianism is associated with greater openness and empathy. The values of vegetarians are based more on universalism, hedonism, stimulation, and self-direction, whereas the values of omnivores are based more on the idea of power. To answer a narrowly defined and clear question, issues such as animal ethics, animal rights, and environmental protection are not considered in this review. The findings of this review, showing marked differences in personality correlating to the choice of diet and the increasing influence of plant-based diets on a global level, indicate that further studies about vegetarianism are warranted.

## Background

As a form of nutrition, vegetarianism describes the partial or complete omission of various animal products. There are different subgroups (e.g., ovo-lacto vegetarianism and veganism) with partially inconsistent definitions (Beardsworth and Keil, 1991, 1992; Ruby, 2012; Rothgerber, 2015b). Often, and in this review, vegetarianism is defined as the abandonment of all meat and seafood products without exception (Craig and Mangels, 2009). There are other nutritional forms that occupy an intermediate position between omnivorism and vegetarianism. Thus, numerous people describe themselves as flexitarians (those who reduce their consumption of meat with the quality of food playing an important role) (Dagevos and Voordouw, 2013), semi-vegetarians (those who exclude red meat), or pescatarians (those who exclude meat and meat products but eat fish and seafood) (Deutsche Gesellschaft für Ernährung e.V, 2013).

In recent years, and especially in Western countries, vegetarianism has gained increasing widespread attention in medical, ecological, political, and other contexts (Key et al., 2006; Baron, 2013; Orlich et al., 2013; Leitzmann, 2014; Bündnis 90/Die Grünen, 2017; Christoffer et al., 2017; Schweizerische Vereinigung für Vegetarismus, 2017). As such, the proportion of vegetarian people is growing noticeably (APF-VVSQ, 2010; Vegetarierbund Deutschland, 2017; Šimčikas, 2018; Vegetarian Society, 2018). Currently, there are ~7.3 million vegetarians in the US (3.2% of the population) (Vegetarian Times, 2008). A more recent source indicates that ~5% of Americans are vegetarians (Newport, 2012; Statista, 2020). In Germany, there are ~8 million (10% of the German population) vegetarians (Vegetarierbund Deutschland, 2017).

The reasons for adopting a vegetarian diet in the Western world are especially related to health, ethics, and morality (Janda and Trocchia, 2001; Fox and Ward, 2008; Bobic et al., 2012; Ruby, 2012; Hoffman et al., 2013; De Backer and Hudders, 2014). In contrast, the motivations for following a vegetarian way of life in so-called “newly industrialized countries” are, above all, based on religion and are culturally rooted (Preece, 2009; Ruby et al., 2013). In India for example, approximately 20–42% of the population follow a vegetarian diet (Yadav and Kumar, 2006; Ruby et al., 2013; Vegetarierbund Deutschland, 2017). A decisive reason for this is Hinduism, the faith to which ~81% of the population belongs (Albrecht et al., 2017).

Many studies have dealt with these different motivational issues (Mathieu and Dorard, 2016; Rosenfeld and Burrow, 2017b), and the medical examinations of various types of diets that have been conducted in many studies indicate that a well-planned vegetarian diet offers a diverse range of health benefits (Craig and Mangels, 2009; Craig, 2010; Baron, 2013; Orlich et al., 2013; Dinu et al., 2017).

Considering the different motives for adopting a vegetarian diet, vegetarianism is not just an eating behavior, but often part of a way of life. A critical reflection on our eating and consumption behaviors and their associated global problems may cause behavioral changes (e.g., becoming a vegetarian) that could contribute to the solving of current global challenges in terms of the climate crisis, habitat destruction, and others (ProVeg International, 2021). Thus, comparisons of different dietary groups could be relevant to find ways to reduce global meat and other animal-based product consumption. However, these changes can be in conflict with culturally conditioned, long-standing lifestyles that often go hand in hand with certain levels of social prosperity. This results in a potential for social conflict. For example, vegetarians and vegans are still often described as a dominant, intolerant, totalitarian, missionary, and militant (Taufen, 2011; Grau, 2014; Gross, 2017; Herries, 2017; Sotscheck, 2018). In contrast, after a comparatively short research span in this scientific field, the impression arises that vegetarians seem to be more empathetic and less interested in social hierarchical structures and seem to have more altruistic values compared to omnivores (Mitte and Kämpfe-Hargrave, 2007; Filippi et al., 2010; Ruby, 2012; Rothgerber, 2015a). For these reasons, it is important to examine the current evidence in detail and to conduct a comprehensive search within the framework of this systematic review.

This article systematically reviews previous studies that compared omnivores, flexitarians, and semi-vegetarians with vegetarians (including vegans) by rating the differences in their personality profiles, values, and empathy skills. For this purpose, all studies that met the inclusion criteria listed in section **Eligibility Criteria** and had target outcomes that raised personality traits, value concepts, and/or the ability to be empathetic were considered for inclusion.

## Methods

### Eligibility Criteria

The present review was conducted according to the guidelines for meta-analyses and systematic reviews of observational studies known as “MOOSE” (meta-analysis of observational studies in epidemiology; 35). Studies were included in this review based on the following criteria:

a) Types of studies: quantitative, epidemiological, observational studies with a cross-sectional design, and cohort studies were eligible. There were no restrictions on language or year of publication.
b) Types of participants: Studies with adult participants were included (no children or adolescents). Additionally, studies needed to compare individuals following an omnivore, flexitarian, or semi-vegetarian diet with individuals following a vegetarian (ovo-lacto vegetarian or vegan) diet. A vegetarian diet was defined as the absence of the consumption of meat, meat products, fish, and seafood. There were no restrictions regarding the time-period over which participants followed the stated diet.
c) Types of outcomes: Studies were eligible if they compared one (or more) of the above-mentioned dietary group(s) with a vegetarian group and assessed at least one of the following: personality characteristics, value patterns, and/or empathetic ability. For this review, personality was defined according to Carver and Scheier (2011) as “a dynamic organization, inside the person, of psychophysical systems that create the person's characteristic pattern of behavior, thoughts, and feelings.” Personality characteristics were defined as continuous variables that differ between persons and stay, more or less, stable over longer periods of time. Other characteristics that can be manipulated by situational factors or changed through therapeutic interventions, such as food neophobia (Pliner and Hobden, 1992), were not defined as personality traits in this review, although this is discussed controversially. Values were defined as “(a) concepts or beliefs, (b) about desirable end states or behaviors, (c) that transcend specific situations, (d) guide selection or evaluation of behavior and events, and (e) are ordered by relative importance” (Schwartz and Bilsky, 1987). Definitions were agreed upon by all researchers.

Qualitative papers, commentaries, and other reviews were ineligible.

### Search Methods

The last update of the complete study search took place on April 19, 2021, with no restrictions on publication year. The search was conducted in three electronic databases (PubMed, Scopus, and PsycINFO).

The search was constructed around the following search terms and is exemplarily shown for the PubMed search:

#1 (“Diet, Vegetarian”[Mesh] OR Vegetarian^*^[Title/Abstract] OR Vegan^*^[title/Abstract] OR “meat-free”[Title/Abstract])
#2 (“Empathy”[Mesh] OR Empath^*^[Title/Abstract] OR Compassion^*^[Title/Abstract])
#3 (“Personality”[Mesh] OR Personality[Title/Abstract] OR“Big Five”[Title/Abstract]OR Trait^*^[Title/Abstract]OR openness[Title/Abstract] OR conscientiousness[Title/Abstract]OR extraversion[Title/Abstract]OR introversion[Title/Abstract]OR agreeableness[Title/Abstract]OR neuroticism[Title/Abstract])
#4 (Value^*^[Title/Abstract]OR “Ethics”[Mesh] OR “Morals”[Mesh] OR “Ethic^*^”[Title/Abstract]OR “Moral^*^”[Title/Abstract])
#5 (#2 OR #3 OR #4)
#6 (#1 AND #5)
#7 (animals[Mesh] NOT humans[Mesh])
#8 (#6 NOT #7)

This search strategy was adapted for each database.

### Data Extraction and Management

First, all the studies from the searches were classified by the first author of this review as either thematically appropriate or not by reading titles and abstracts. To improve the quality of the screening stage, 5% of all titles and abstracts were randomly screened by a second author for independent agreement purposes, where there was complete agreement. Thereafter, the thematically appropriate studies were either included according to their full texts or excluded due to the characteristics of their participants, outcomes, or design. Potentially eligible studies were examined independently by three authors and decisions concerning the inclusion or exclusion of studies were made collaboratively.

### Risk of Bias in Individual Studies

The risk of bias was assessed by two authors independently, using the Newcastle–Ottawa scale (NOS) for case-control studies (Wells et al., 2014). The absence of an exposure factor or a disease in the inherent question for this review and, accordingly, in the included studies, necessitated the use of a shortened variant of the NOS. Thus, the category “exposure” and the subcategory “definition of controls” (in the category “selection”) were excluded. The following categories were examined: “selection” [with the subcategories: “adequate case definition,” “representativeness of the cases,” and “selection of controls” (in each subcategory, a maximum of one star could be awarded)] and “comparability” [with the subcategory “comparability of cases and controls based on the design or analysis” (with a maximum of two stars to rating]) (ClinicalTrials.gov, 2017).

As an exemplary explanation of the procedure and the requirements for the distribution of stars, a transparent presentation of the methods of the individual studies and adequate definitions were emphasized. “Representativeness” was rated with a star if the appropriate nutrition groups were recruited adequately. Stars for the “comparability” of nutritional groups were given when disruptive factors such as age and sex were examined.

Finally, a maximum of five stars could be achieved. A pre-existing rating system was used to classify the studies into corresponding quality grades (Penson et al., 2012). It was adjusted due to the reduction of the maximum number of achievable stars from nine to five stars (^*^) as follows:

a) Good quality (5 to 3^*^): 2 or 3 stars in the selection AND 1 or 2 stars in the comparability domain
b) Fair quality (4 to 2^*^): 1 or 2 stars in the selection AND 1 or 2 stars in the comparability domain
c) Poor quality (1 to 0^*^): 0 or 1 star in selection OR 0 stars in the comparability domain

No studies were excluded from the review due to a high risk of bias.

## Results

### Search

**Figure 1:**
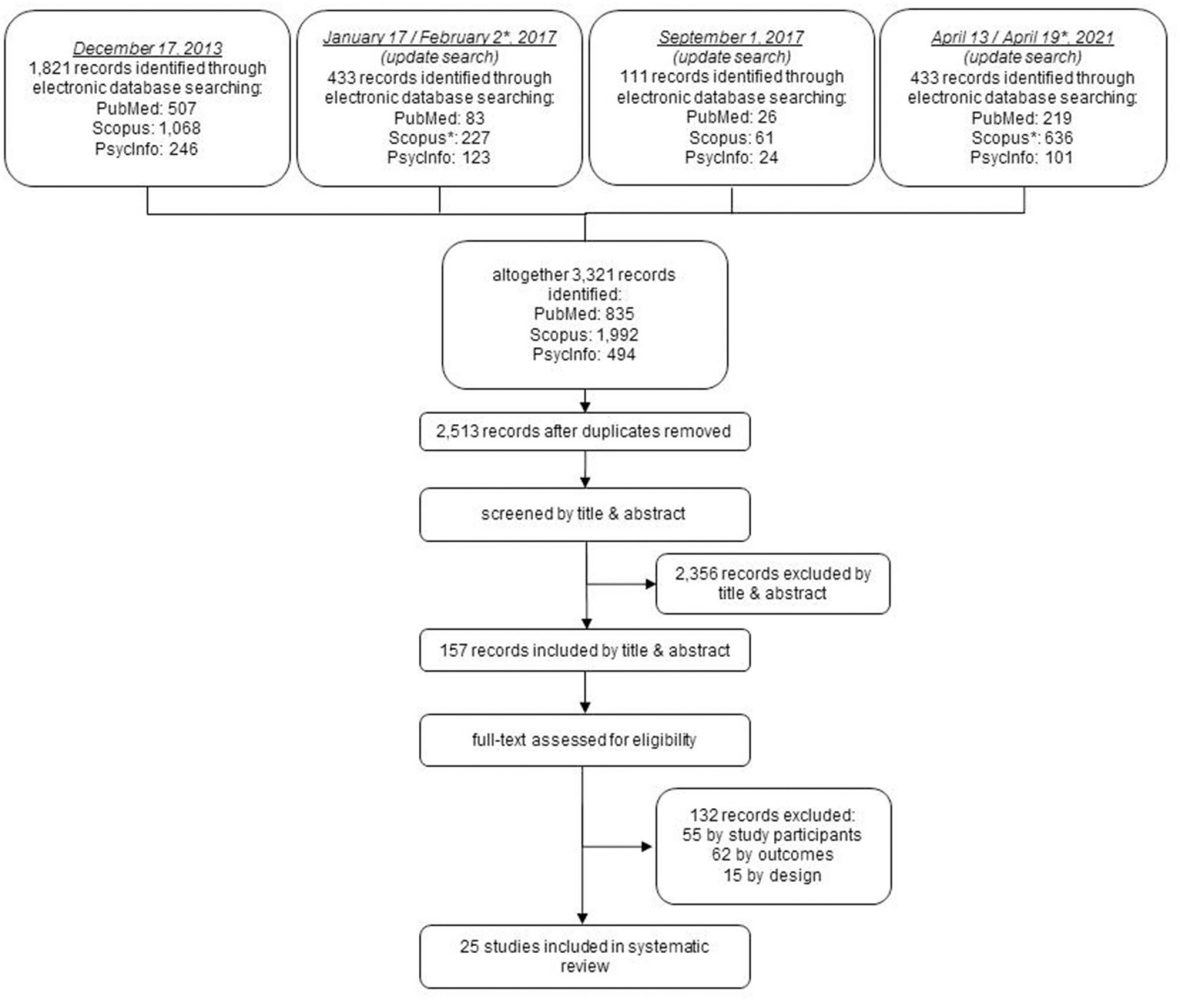
Flowchart of results of the search.

The search was conducted over four separate periods of time. A time filter (the date of the previous search until the day of the occurring search) was applied to the three update searches. Thus, it was ensured that the status of the study was current. The search generated a total of 3,321 records, of which 808 were duplicates and were excluded (Figure 1). After investigating titles and abstracts, the remaining 157 records were assessed for eligibility by studying their full texts. One hundred and thirty-two records were excluded based on not meeting all the above-mentioned eligibility criteria (55 by study participants, 62 by outcomes, and 15 by design). Finally, 25 appropriate studies were included in the review, published between 1978 and 2021. Of these 25 studies, six contained two suitable sub-studies each. The latter are partly presented separately in the presentation of results (then mentioned explicitly). If these sub-studies are considered individually, a total of 31 studies were included in this review.

### Study Characteristics

The characteristics of each study and its participants, outcome measures, and results, are presented in Table 1.

**Table 1:** Characteristics of the included studies.

**References**	**Origin of study; setting**	**Study design**	**Participants**	**Sample size (*n*)**	**Sample characteristics (mean age; female %; ethnicity/population group)**	**Outcome measures**	**Results**
Allen et al. (2000)	Australia, New Zealand; metropolitan region	Cross-sectional study	*Substudy 1:* omni., pesc., ovo-lacto veg., vegans *Substudy 2:* as in substudy 1	*Substudy 1:* *n* = 158 (no further info.) *Substudy 2:* *n* = 378 (324 omni., 54 veg.)	*Substudy 1:* 46 y; female 51%; 65% Pākehā *Substudy 2:* 36 y; female 55%; 90% Pākehā	*Substudy 1:* **Personality:** RWA *via* RWAS Altemeyer, 1981, SDO *via* SDOS Pratto et al., 1994 *Substudy 2:* **Values:** human values *via* Rokeach Value Survey + 4 values Rokeach, 1973	*Substudy 1:* **Personality:** Omnivorism was associated with greater RWA (*p* < 0.05), SDO (*p* < 0.05); vegetarianism/veganism with lower RWA (*p* < 0.05), SDO (*p* < 0.05). *Substudy 2:* **Values:** Omnivorism was associated with greater emphasis on self-control (*p* < 0.001), responsibility (*p* < 0.05), logic (*p* < 0.05), equity (*p* < 0.05), social power (*p* < 0.05). Vegetarianism/veganism was associated with greater emphasis on intellectualism (*p* < 0.001), excitement (*p* < 0.001), love (*p* < 0.05), happiness (*p* < 0.05), growth (*p* < 0.05), peace (*p* < 0.001), equality (*p* < 0.001), social justice (*p* < 0.05).
Bilewicz et al. (2011)	Poland, Germany; internet	Cross-sectional study	*Substudy 3:* Omni., veg. (ovo-lacto veg., vegans)	*Substudy 3:* *n* = 325 (148 omni., 177 veg.)	*Substudy 3:* 30.08 y; female ≈ 78%; no info.	*Substudy 3:* **Personality:** SDO *via* SDOS Pratto et al., 1994	*Substudy 3:* **Personality:** Vegetarians had lower scores on the SDOS than omnivores (*p* < 0.001).
Cliceri et al. (2018)	Italy; internet *(blogs, social networks, emails)*, pamphlet distribution, word of mouth in urban region, social environments attended by veg.	Cross-sectional study	omni., flex., veg.	*n = 125* (39 omni., 55 flex., 31 veg.)	28.6 y; female 72.8%; no info.	**Personality:** Food Neophobia Scale (FNS) Pliner and Hobden, 1992; Laureati et al., 2018, Pathogen Disgust (PD), Moral Disgust (MD) *via* Three-Domain Disgust Scale (TDDS) Tybur et al., 2009 **Empathy:** Perspective-Taking (PT), Fantasy (FS), Empathic Concern (EC), Personal Disease *via* Interpersonal Reactivity Index Davis, 1983; Albiero et al., 2006	**Personality:** On average, all three dietarian groups were low in neophobia with no significant difference in the FNS (*p =* 0.112). Compared to vegetarians, omnivores and flexitarians had a significant higher PD toward infectious agents (*p =* 0.003) and tendentially higher MD toward antisocial activities (not significant with *p =* 0.074). **Empathy:** All three dietarian groups tendentially had high scores of cognitive and emotional empathy (Personal Disease excluded). Vegetarians scored significantly higher on PT (*p =* 0.035) in comparison to omnivores. Without significance, vegetarians scored higher than omnivores for EC (*p =* 0.085). The values of flexitarians for PT and EC were between those of omnivores and vegetarians. No effect was found between all groups for FS (*p =* 0.559) and Personal Disease (*p =* 0.333).
Cruwys et al. (2020)	Australia (origin of 4 from 5 authors and seat of the ethics committee) but no clear info.; Internet *(forums, social media, special interest groups)*, snowball sampling, university recruitment pool	Cross-sectional	omni. (paleo, gluten free, weight loss), veg., vegan	*n =292* (116 omni. with 42 paleo and 38 gluten free and 36 weight loss, 48 veg., 128 vegan)	31.44 y; female 85.5%; 84.6% Caucasian	**Personality:** self-control *via* Brief Self-Control Scale Tangney et al., 2004, emotional eating *via* Dutch Eating Behavior Questionnaire Wardle, 1987,extraversion, agreeableness, conscientiousness, neuroticism, openness to experience *via* 20-item Mini-IPIP Donnellan et al., 2006 (a short version of the Five-Factor Model questionnaire Goldberg, 1999), self-efficacy *via* a validated single-item scale Hoeppner et al., 2011 **Values:** care, purity, loyalty, fairness, authority *via* moral foundation questionnaire Graham et al., 2011; Davies et al., 2014	**Personality:** Vegans were the dietary group least likely to state the facilitator of conscientiousness and a lack of willpower as a barrier to adherence. They did not base their diet on an individual context and most frequently stated that they felt no barriers at all, followed by the vegetarian group. A lack of willpower as a barrier to adherence was mentioned second mostly by the paleo group and most often by weight loss dieters as well as mood/emotion. Inconvenience as a barrier was reported most frequently by people on a gluten-free dietary. **Values:** Vegans were the dietary group most likely and vegetarians who were second most likely to state the adherence facilitators of ethical/moral concerns and identity. Vegetarians least frequently stated health as a facilitator. The paleo group most likely reported of enjoyment and second most likely reported of health as a facilitator. Health as a barrier was reported most frequently by people on a gluten-free dietary.
De Backer and Hudders (2015)	Belgium; internet *(university lists, social media, vegetarian organization “Ethisch Vegetarisch Alternatief”)*	Cross-sectional study	omni., flex., veg.	*n* = 299 (≈ 90 omni., ≈ 83 flex., ≈ 126 veg.)	34.4 y; female 62%; Fleming	**Values:** attitudes toward human welfare, moral issues *via* Moral Foundations Questionnaire-30 Graham et al., 2011	**Values:** Compared to flexitarians, vegetarians believed more in the importance of avoidance of human suffering (*p* < 0.001). Compared to flexitarians, omnivores believed more in the importance of respect for status (authority/respect) (*p* < 0.001).
Dhar et al. (2008)	India; no info.	Cross-sectional study	omni. (“non-veg.”) teachers and medical doctors, veg. teachers and medical doctors	*n* = 72 (23 omni.; 49 veg. with 23 of 33 teachers & 26 of 39 medical doctors)	40.5 y; no info.; no info.	**Values:** existing values, ought-to-be values *via* Checklist of Values Dhar, 1996	**Values:** *existing values*: Compared to omnivores, vegetarians (irrespective of profession) perceived discipline. *ought-to-be values*: Compared to omnivores, vegetarians (irrespective of profession) perceived wealth. Compared to vegetarians, omnivores (irrespective of profession) perceived benevolence.
Forestell et al. (2012)	U.S.A.; college *(students)*	case-control study, conveniance sample	omni., semi-veg., flex., pesc., veg. (ovo-lacto veg., vegans)	*n* = 240 (91 omni., 29 semi-veg., 37 flex., 28 pesc., 55 veg.)	omni.: 19.10 y, semi-veg.: 19.62 y, flex.: 18.51 y, pesc.: 19.75 y, veg.: 19.42 y; female 100%; 79% Caucasian, 12% Asian, 6% African American, 3% other	**Personality: five** dimensions *via* NEO Five-Factor Inventory McCrae and Costa, 2004, general neophobia *via* General Neophobia Scale Pliner and Hobden, 1992, variety seeking *via* Variety Seeking Scale Van Trijp and Steenkamp, 1992	**Personality:** Vegetarians were significantly more open to new experiences, variety seeking than omnivores (all *p* < 0.05; *ps* < 0.012).
Forestell and Nezlek (2018)	U.S.A.; university undergraduates in psychology classes	Case-control study, Conveniance sample	omni., semi-veg. (incl. pesc.), veg., vegans	*n = 6422* (4.955 omni.; 1,191 semi-veg. with 153 pesc. and 158 semi-veg. and 880 occasional omni.; 276 veg. with 194 lacto-ovo-veg. and 38 lacto-veg. and 44 vegans)	18.96 y; female ≈ 57.7% (omni. female ≈ 51.2%, semi-veg. female ≈ 81.0%, veg. female ≈ 73.9%); 67.7% White, 8.1% Black, 11.7% Asian, 12.6% others	**Personality:** agreeableness, extraversion, conscientiousness, neuroticism, openness to experience *via* Big Five Inventory (BFI-44) John et al., 1991,	**Personality:** Vegetarians were more open than semi-vegetarians (*p =* 0.001) and semi-vegetarians were more open than omnivores (*p =* 0.001). Omnivores were less neurotic than vegetarians (*p* < 0.001) and semi-vegetarians (*p* < 0.001); no difference was found between vegetarians and semi-vegetarians (*p =* 0.98). There were no significant differences between the dietary groups in agreeableness, extraversion and conscientiousness.
Hopwood and Bleidorn (2020)	*Substudy 2:* U.S.A.: online data collection platform *(Prolific data collection service: https://www.prolific.co)*	Cross-sectional study	*Substudy 2:* omni., veg.	*Substudy 2:* *n = 682* (431 omni., 251 veg.)	*Substudy 2:* 31.04 y, female ≈ 61.9%; ≈ 72,6% White, ≈ 5.9% Black, ≈ 12.0% Asian, ≈ 0.4% Pacific Islander, ≈ 6.3% multiracial, ≈ 2.8% other races, ≈ 8.5% Latinx	*Substudy 2:* **Personality:** **antisocial personality features* via* 60-item version of the International Personality Item Pool Maples-Keller et al., 2019 with neuroticism, extraversion, agreeableness, and conscientiousness, Brief Personality Inventory for DSM-5 (American Psychiatric Association, 2013) with negative affectivity, detachment, psychoticism, antagonism, and disinhibition, callousness and selfcenteredness scales from the Elemental Psychopathy Assessment Lynam et al., 2011, entitlement, indifference (, and lack of empathy) scales from the Five Factor Narcissism Inventory Glover et al., 2012 for maladaptive antagonism facets, SDO *via* SDOS Pratto et al., 1994 **Empathy: (**entitlement, indifference, and) lack of empathy scale(s) from the Five Factor Narcissism Inventory Glover et al., 2012 for maladaptive antagonism facets	*Substudy 2:* **Personality:** In comparison to vegetarians, omnivores were less agreeable and more callous, self-centered, entitled, and indifferent. Omnivores had higher scores in SDO and in agentic values than vegetarians. **Empathy**: In comparison to vegetarians, omnivores were less empathetic.
Kalof et al. (1999)	U.S.A.; telephone interviews	Cross-sectional study	omni., veg.	*n* = 420 (398 omni., 22 veg.)	44.2 y; female 56%; 83.3% Caucasian, 6.5% African-American	**Values:** environmental values *via* modification of Schwartz' Value Survey Schwartz, 1992	**Values:** Vegetarianism was significantly positively correlated with altruistic values (*p* < 0.01) and negatively correlated with traditional values (*p* < 0.05).
Kessler et al. (2018)	Germany; Paper–pencil questionnaire at “VegMed 2013” conference in Berlin	Cross-sectional study	omni., veg., vegan Medical professionals	*n = 197* (55 omni., 78 veg., 64 vegans)	omni.: 42.8 y, veg.: 38.3 y, vegans: 37.3 y; omni. female 80.0%, veg. female 75.6%, vegan female 76.6%; no info.	**Personality:** Big Five SOEP Inventory Lang et al., 2011 **Values:** Portraits Value Questionnaire (21 Item-Version; Schmidt et al., 2007), WHO Quality of Life-BREF (WHOQOL-BREF; World Health Organization, 2014 **Empathy:** Empathizing Scale (Short Form; Samson and Huber, 2010)	**Personality, values, empathy:** In all measures of personality, values, empathy, no statistically significant differences between omnivorous, vegetarian and vegan medical professionals were found.
Lindeman and Sirelius (2001)	Finland; Majority college *(students)* and employees	Cross-sectional study	*Substudy 1:* omni., omni. Fat/cholesterol avoiders (FCAs), pesc., veg. *Substudy 2:* omni., pesc., veg.	*Substudy 1:* *n* = 82 (≈ 36.4 omni. with ≈ 16.6 omni. and ≈ 19.8 omni. FCAs, ≈ 24.9 pesc. ≈ 20.7 veg.) *Substudy 2:* *n* = 149 (≈ 108 omni., ≈ 25 pesc., ≈ 16 veg.)	*Substudy 1:* 27 y; female 100%; no info. *Substudy 2:* 31.5 y; female 100%; no info.	**Values:** humanism, normativism *via* version of Tomkins' Polarity Scale De St. Aubin, 1996, Food Choice Ideologies (FCIs) as summary patterns of correlations among values and Food Choice Motives, values *via* shortened (substudy 1)/original (substudy 2) Schwartz' Value Survey Schwartz, 1992	**Values:** Vegetarians endorsed EI (universalism, stimulation, self-direction) more than omnivores. EI and PI was positively associated with a humanistic view of the world. HI (tradition, conformity, security) was positively associated with a normative view of the world and was more prominent among fat/cholesterol avoiders than among omnivores/vegetarians.
Nezlek et al. (2018)	U.S.A.; Undergraduates, daily diary for 14 days, online questionnaire	Cross-sectional study, convenience sample	omni., semi-veg. (incl. pesc.), veg.	*n = 403* (323 omni., 56 semi-veg., 24 veg.)	18.8 y; female 62%; no info.	**Personality:** self-esteem *via* four items (adapted for daily use) from a widely used measure of self-esteem Rosenberg, 1965, daily depressogenic thinking *via* three items based on Beck's Cognitive Triad Beck, 1967, self-focused attention (reflection, rumination) *via* items based on Rumination-Reflection Questionnaire Trapnell and Campbell, 1999, daily affect based on circumplex model of emotions (e.g., Feldman Barrett and Russell, 1998) **Values:** daily life satisfaction *via* two items based on those used by Oishi et al. (2007), presence of meaning in life *via* two items that had been used in previous diary studies (e.g., Kashdan and Nezlek, 2012)	There were no significant outcome differences between omnivores and semi-vegetarians. **Personality:** Compared to omnivores and semi-vegetarians, vegetarians had lower self-esteem, lower psychological adjustment, stronger negative moods, more negative social events, and had more pronounced thoughts about themselves. **Values:** In comparison to omnivores and semi-vegetarians, vegetarians reported lower satisfaction with daily life with marginally significance (*p* < 0.10).
Pfeiler and Egloff (2018)	Substudy 1: German private households: German Socio-Economic Panel (SOEP-CORE; Wagner et al., 2007) 2013, 2014, Innovation Sample of the SOEP (SOEP-IS; Schupp et al., 2016)	*Substudy 1:* longitudinal representative survey	*Substudy 1:* veg. (incl. vegans)	*Substudy 1:* *n = 4496* (4373 omni., 123 veg. incl. 13 vegans)	*Substudy 1:* 51.84 y; female 52.3% (73.17% of the veg.); no info.	*Substudy 1:* **Personality:** openness, conscientiousness, extraversion, agreeableness, neuroticism *via* 15-item German short version of the Big Five Inventory (BFI-S; Gerlitz and Schupp, 2005; see Hahn et al., 2012), trust *via* three items based on the General Social Survey (GSS) and the World Values Survey (WSS) + 1 item (Dohmen et al., 2008), patience and impulsivity Vischer et al., 2013, risk aversion *via* 11-point scale Kahneman and Tversky, 1979; Dohmen et al., 2011, optimistic attitude about the future *via* 1 item Trommsdorff, 1994 **Values:** political attitudes *via* conservatism, level of political interest, current life-satisfaction Schimmack et al., 2009, satisfaction with health	*Substudy 1:* **Personality:** In comparison to omnivores, vegetarians scored significantly higher in openness, trust and significantly lower in consciousness. There were no longer significant differences between the two diet groups in trust after controlling for socio-demographic variables. No other significant differences were found for any of the other variables. **Values:** In comparison to omnivores, vegetarians scored significantly higher in interest in politics and significantly lower in conservativism. No significant differences were found for current life-satisfaction and satisfaction with health for the different dietarian groups.
Piazza et al. (2015)	United Kingdom, U.S.A., Australia; *Substudy 2:* uni. campus *Substudy 4:* internet *(MTurk Amazon Mechanical Turk, 2017)*	Cross-sectional study	*Substudy 2:* omni., semi-veg./pesc., veg. (ovo-lacto veg., vegans) *Substudy 4:* omni., semi-veg./pesc., ovo-lacto veg., vegans	*Substudy 2:* *n* = 171 (73 omni., 40 semi-veg./pesc., 58 veg.) *Substudy 4:* *n* = 215 (57 omni., 90 semi-veg./pesc., 44 ovo-lacto veg., 24 vegans)	*Substudy 2:* 22.91 y; female ≈ 62%; no info. *Substudy 4:* 31.89 y; female ≈ 55.3%; no info.	*Substudy 2:* **Personality:** SDO *via* SDOS Pratto et al., 1994 *Substudy 4:* **Values:** pride, guilt, discomfort, moral self-regard (all related to consumption and use of animal products)	*Substudy 2:* **Personality:** Omnivores endorsed exploitative ideologies more than semi-vegetarians/pescetarians (*p* < 0.001) and vegetarians (*p* < 0.001); no significant difference between the last two. *Substudy 4:* **Values:** There are indications that, in comparison to ovo-lacto vegetarians and vegans, omnivores more often experienced less pride and moral self-regard (related to their consumption and use of animal products).
Preylo and Arikawa (2008)	U.S.A.; Supermarkets	Cross-sectional study	omni (“non-veg.”), veg. (ovo-lacto veg., vegans, fruitarians)	*n* = 139 (67 omni., 72 veg.)	32.4 y; female ≈ 63.3%; no info.	**Empathy:** Perspective-Taking (PT), Fantasy (FS), Empathic Concern (EC), Personal Distress (PD) *via* Interpersonal Reactivity Index Davis, 1983	**Empathy:** Vegetarians scored significantly higher than omnivores on the EC, FS, PT subscales (*p* < 0.001), and the PD subscale (*p* < 0.01). EC and PT were the strongest predictors of vegetarian diet.
Rosenfeld and Burrow (2018)	*Substudy 3:* (Northeastern of the) U.S.A., undergraduates	*Substudy 3:* Cross-sectional study	*Substudy 3:* omni., veg.	*Substudy 3:* *n = 353* [305 omni., 48 veg. (incl. vegans)]	*Substudy 3:* 20.39 y; female 78%; no info.	*Substudy 3:* **Personality:** strictness *via* self-developed Dietarian Identity Questionnaire (DIQ) – based on Rosenfeld and Burrow (2017a) Unified Model of Vegetarian Identity	*Substudy 3:* **Personality:** Compared to omnivores, vegetarians scored significantly higher in strictness.
Rothgerber (2015a)	U.S.A.; Internet *(Survey Monkey®* SurveyMonkey, 2017 *veg. oriented online-groups)*	Cross-sectional study	flex., ovo-lacto veg., vegans	*n* = 556 (143 flex., 206 ovo-lacto veg., 207 vegans)	36.44 y; female 76%; 81% U.S.A., 14% Canada, 5% another country	**Personality:** absolutism (in strictly following diet), guilt over violating diet (ethical, health concerns)	**Personality:** In comparison to ovo-lacto vegetarians and vegans, flexitarians scored significantly lower on absolutism (in strictly following their diet), reported violating their diet more and felt less ethically associated guilt when doing so (all at *p =* 0.000). Vegans displayed greater ethical guilt than did ovo-lacto vegetarians (*p =* 0.034).
Rothgerber (2015b)	U.S.A.; Internet *(MTurk Amazon Mechanical Turk, 2017)*	Cross-sectional study	flex., ovo-lacto veg., vegans	*n* = 196 (109 flex., 70 ovo-lacto veg., 17 vegans)	35.37 y; female 63%; U.S.A. as country of origin	**Personality:** misanthropy Wuensch et al., 2002 **Values:** ethical idealism *via* Ethics Position Questionnaire Forsyth, 1980	**Personality:** In comparison to ovo-lacto vegetarians, flexitarians scored significantly lower on misanthropy. **Values:** In comparison to vegans, flexitarians and ovo-lacto vegetarians scored significantly lower on idealism.
Ruby et al. (2013)	U.S.A., Canada, India *Substudy 1:* Internet *(MTurk Amazon Mechanical Turk, 2017)* *Substudy 2:* Euro-Canadians: uni., online veg. group Euro-Americans: online veg. group, internet *(MTurk Amazon Mechanical Turk, 2017)* “Mturk Indians”: internet *(MTurk Amazon Mechanical Turk, 2017)* “Karantaka Indians”: uni.	Cross-sectional study	*Substudy 1:* omni., veg. *Substudy 2:* omni., veg.	*Substudy 1:* *n* = 272 (159 Euro-Americans: 145 omni., 14 veg.; 113 Indians: 66 omni., 47 veg.) *Substudy 2:* *n* = 828 (106 Euro-Canadians: 91 omni., 15 veg.; 266 Euro-Americans: 245 omni., 21 veg.; 256 “Mturk Indians”: 184 omni., 72 veg.; 200 Karnataka Indians”: 96 omni., 104 veg.)	*Substudy 1:* Euro-Americans (≈ 58%): 36.6 y; female 65%; see above Indians: 29.1 y; female 40%; see above *Substudy 2:* Euro-Canadians: 25.4 y; female 60%; see above Euro-Americans: 35.7 y; female 64%; see above “Mturk Indians”: 29.3 y; female 33%; see above “Karnataka Indians”: 25.4 y; female 51%; see above	*Substudy 1:* **Personality:** RWA *via* RWAS Altemeyer, 1981 **Values:** *via* Portrait Value Questionnaire Schwartz et al., 2001 with (here) focus on universalistic values *Substudy 2:* **Values:** considerations associated with the Five Moral Foundations (from Graham et al., 2009): purity, authority, ingroup, harm, fairness	*Substudy 1:* **Personality:** Vegetarians scored significantly lower on RWA than omnivores (*p* < 0.001); significant among Euro-Americans (*p* < 0.004). **Values:** Vegetarians scored significantly higher on Universalism than omnivores (*p* < 0.001); significant just among Euro-Americans (*p* < 0.005). *Substudy 2:* **Values:** Vegetarians endorsed the ethic of purity significantly more than omnivores (*p* < 0.001); significant just among Indians (all *p* < 0.001). Indian vegetarians endorsed the ethic of authority significantly more than omnivores (Mturk: *p* < 0.01, Karnataka: *p* < 0.001); among Euro-Americans, vegetarians endorsed it less than omnivores (*p =* 0.07); no significant differences among Euro-Canadian dietary groups. Vegetarians endorsed the ethic of harm significantly more than omnivores (*p* < 0.001); significant among Indians (all *p* < 0.001), marginally significant among Euro-Americans (*p* < 0.06). Indian and Euro-American vegetarians endorsed the ethic of fairness significantly more than omnivores (Indians: *p* < 0.001, Euro-Americans: *p* < 0.03).
Sariyska et al. (2019)	Germany, Ulm; mostly psychology classes Uni. Ulm - internet *Substudy 1:* Ulm Gene Brain Behavior Project, Dark Triad traits and Internet Use Disorders (Sindermann et al., 2018) *Substudy 2:* Dark Triad traits and Internet Use Disorders (Sindermann et al., 2018)	Cross-sectional study	*Substudy 1:* omni., veg. (incl. vegans) *Substudy 2:* omni., veg. (incl. vegans)	*Substudy 1:* *n = 1140* [1009 omni., 131 veg. (incl. vegans)] *Substudy 2:* *n = 444* [389 omni., 55 veg. (incl. vegans)]	*Substudy 1:* 23.54 y; female ≈ 68.7%; no info. *Substudy 2:* 30.12 y; female ≈ 70.3%; no info.	**Personality:** six primary emotional systems (seeking, play, care, fear, anger, sadness + spirituality) *via* Affective Neuroscience Personality Scales (ANPS) (Davis et al., 2003; German version by Reuter et al., 2017), Machiavellianism, non-pathological narcissism, non-pathological psychopathy *via* The Short Dark Triad Scale (SD3; Jones and Paulhus, 2014)	*Substudy 1:* **Personality:** In comparison to omnivores, vegetarians had significantly higher scores in care, sadness, spirituality and lower scores in play, the latter was no longer significant after Bonferroni correction. Machiavellianism, non-pathological narcissism, and non-pathological psychopathy were higher in omnivores than in vegetarians without reaching significance. *Substudy 2:* **Personality:** Machiavellianism (*p* < 0.001), non-pathological narcissism (*p* < 0.001), non-pathological psychopathy (*p =* 0.007) were significantly higher in omnivores than in vegetarians. After consideration of sex, only the mentioned differences in Machiavellianism and narcissism stayed significant.
Sims (1978)	U.S.A.; Uni. campus	Cross-sectional study	omni (“non-veg.”), veg.	*n* = 487 (385 omni., 102 veg.)	21 y; female ≈ 66.7%; 95% caucasian	**Personality:** social desirability Crowne and Marlowe, 1950 **Values:** value-orientations toward the use of food, nutrition is important attitude (Sims, unpublished data)	**Personality:** No significant differences were shown on the social desirability measurement. **Values:** Compared to omnivores, vegetarians scored significantly higher on food-related value-orientations of ethics, health, religion (*p* < 0.001) and education (*p* < 0.05). The scale of food-related value-orientations of ethics was most positively related to vegetarianism. Compared to vegetarians, omnivores scored significantly higher on food-related value-orientations of economics (*p* < 0.01), familism (*p* < 0.05) and social/psychological uses of food (*p* < 0.01). The scale of food-related value-orientations of social/psychological uses of food was most negatively associated with vegetarianism. No significant differences on food-related value-orientations of aesthetic and creativity. Compared to vegetarians, omnivores scored slightly higher on the nutrition is important attitude (*p* < 0.05).
Tan et al. (2021)	*Substudy 1a:* New Zealand; Daily Life Study: daily life of uni. students, e.g. from psychology classes 2013-2014 *Substudy 1b:* New Zealand; uni. Students and U.S.A.; online *[MTurk (Amazon Mechanical Turk, 2017)]*; 2017-2019	Cross-sectional study	*Substudy 1a:* omni., restricted omni. (excluded either red meat, poultry, or fish), veg*ns (veg., vegans) *Substudy 1b:* omni., restricted omni. (excluded either red meat, poultry, or fish), veg*ns (veg., vegans)	*Substudy 1a:* *n = 797* (766 omni. with 645 omni. and 121 restricted omni., 31 veg*ns with 25 veg. + 6 vegans) *Substudy 1b:* *n = 1534 with 28% from New Zealand, 72% from U.S.A*. (1427 omni. with 1227 omni. and 200 restricted omni., 107 veg*ns with 64 veg. + 43 vegans)	*Substudy 1a:* 19.72 y; female 73%; no info. *Substudy 1b:* 21.90 y; female 69%; no info.	*Substudy 1a:* **Personality:** 60-item NEO Five Factor Inventory (NEO-FFI; Costa and McCrae, 1985), openness, intellect *via* Big Five Aspect Scales (BFAS; DeYoung et al., 2007) *Substudy 1b:* **Personality:** Big Five domains and their aspects (i.e., openness, intellect, withdrawal, volatility, compassion, politeness, industriousness, orderliness, assertiveness, and enthusiasm) *via* 100-item Big Five Aspect Scales (BFAS; DeYoung et al., 2007)	*Substudy 1a:* **Personality:** Veg*ns scored significantly higher on openness/intellect than restricted-omnivores (*p =* 0.007) and omnivores (*p* < 0.001)., d = 0.81. In pairwise comparisons, this difference did not remain significant for intellect. *Substudy 1b:* **personality:** In comparison to omnivores (*p =* 0.001) and restricted-omnivores (*p =* 0.05), veg*ns were significantly higher on compassion in pairwise comparisons. Within the MTurk (U.S.A.) sample, this difference was not significant between veg*ns and restricted-omnivores (*p =* 0.30). Only in comparison to omnivores (*p* < 0.001), veg*ns scored significantly higher on intellect.
Trethewey and Jackson (2019)	Australia; internet *(social network groups, uni. campus sites, snowball sampling)*	Cross-sectional study	omni., veg., vegans	*n = 336* (110 omni., 56 veg., 170 vegans)	28 y; female 79%; no info.	**Values:** personal-health *via* self-designed questionnaire by the authors	**Values:** In personal-health values, omnivores scored significantly lower than vegetarians (*p =* 0.016) and vegans (*p* < 0.001).
Veser et al. (2015)	Germany; internet *(online advertisement, flyers; dietetic interest groups)*	Cross-sectional study	omni., ovo-lacto veg., vegans	*n* = 1381 (478 omni., 434 ovo-lacto veg., 469 vegans)	32 y; female ≈ 71.3%; no info.	**Personality:** tendency to be prejudiced (TP) *via* Motivation for Prejudice-free Behavior Scale (Banse and Gawronski, 2003), RWA Funke, 2003, SDO *via* short form of SDOS Pratto et al., 1994; Von Collani, 2002	**Personality:** Compared to ovo-lacto vegetarians and vegans, omnivores had a significantly higher TP, authoritarianism and SDO scores.

*The terminology used to refer to the various nutritional groups was standardized, since, in some cases, different names were used for the same diet. While some studies covered other data, only outcomes relevant for the research question were included*.

*flex., flexitarian(s); info., information; omni., omnivore(s); pesc., pescetarian(s); RWA(S), Right-Wing Authoritarianism (Scale); SDO(S), Social Dominance Orientation (Scale); Uni., university; veg., vegetarian(s); y, years*.

### Setting and Participant Characteristics

Twenty-five observational studies, with a total of 23,589 participants, were included in this review. All studies obtained information about the diets of participants through self-reporting. Of these, a total of 17,403 participants were omnivores (including paleo, gluten-free, weight loss restricted, and occasional omnivores, omnivore fat/cholesterol-avoiders), 4,117 were vegetarians (vegans included), 427 were flexitarians, and 1,484 were semi-vegetarians or pescatarians. Of the remaining 158 participants, the numerical distribution to the different nutritional groups was not apparent to the researchers (Allen et al., 2000: sub-study 1). Sample sizes ranged from 72 (Dhar et al., 2008) to 6,422 (Forestell and Nezlek, 2018), with a median of 386 (336, respectively, when considering each of the two sub-studies of six studies independently (Allen et al., 2000; Lindeman and Sirelius, 2001; Ruby et al., 2013; Piazza et al., 2015; Sariyska et al., 2019; Tan et al., 2021). The mean age of participants ranged from 18.8 (Nezlek et al., 2018) to 51.84 years (Pfeiler and Egloff, 2018), with a median of 30.9 years (31.04 years, respectively), when considering each of the two sub-studies of six studies independently (Allen et al., 2000; Lindeman and Sirelius, 2001; Ruby et al., 2013; Piazza et al., 2015; Sariyska et al., 2019; Tan et al., 2021). Between 52.25 (Ruby et al., 2013) and 100% (Lindeman and Sirelius, 2001; Forestell et al., 2012) of the participants were female, with a median of 68.1%. When considering each of the two sub-studies of six studies independently (Allen et al., 2000; Lindeman and Sirelius, 2001; Ruby et al., 2013; Piazza et al., 2015; Sariyska et al., 2019; Tan et al., 2021), the percentage of women was between 51 (Allen et al., 2000) and 100% (Lindeman and Sirelius, 2001; Forestell et al., 2012), respectively, with a median of 67.7%. In the study conducted by Dhar et al. (2008), no information on gender distribution was provided. Information on the ethnicity of the participants or population groups differed widely and was often not specified. Most of the studies were conducted in industrialized countries (often in the USA). The participants in two of the studies were partially or completely of Indian origin (Dhar et al., 2008; Ruby et al., 2013).

### Outcome Measures

Personality characteristics were assessed in nine studies. Values were also assessed in nine studies or, respectively, in 10, when considering sub-studies independently. There was only one study that directly assessed empathy.

#### Personality

Three studies collected data about right-wing authoritarianism (RWA), namely, Allen et al. (2000) and Ruby et al. (2013). These three studies used the “Right-Wing Authoritarianism Scale” (Altemeyer, 1981), which Veser et al. (2015) collected *via* Five studies (Allen et al., 2000; Bilewicz et al., 2011; Piazza et al., 2015; Veser et al., 2015; Hopwood and Bleidorn, 2020, short form) investigated social dominance orientation (SDO) using the “Social Dominance Orientation Scale” (Pratto et al., 1994). Furthermore, studies assessed the tendency to be prejudiced through the “Motivation for Prejudice-Free Behavior Scale” (Banse and Gawronski, 2003; Veser et al., 2015), misanthropy (Rothgerber, 2015b), social desirability (Sims, 1978), self-esteem (Nezlek et al., 2018), self-efficacy (Cruwys et al., 2020), and self-control *via* the “Brief Self-Control Scale” (Tangney et al., 2004; Cruwys et al., 2020), and strictness (Rosenfeld and Burrow, 2018), absolutism, and guilt over violating one's diet (Rothgerber, 2015a). The “Big Five” personality traits (openness to experience, conscientiousness, extraversion, agreeableness, and neuroticism) and their aspects were, partly or completely, measured using different questionnaires in seven different studies (Forestell et al., 2012; Forestell and Nezlek, 2018; Kessler et al., 2018; Pfeiler and Egloff, 2018; Cruwys et al., 2020; Hopwood and Bleidorn, 2020; Tan et al., 2021). Forestell et al. (2012) measured variety-seeking behavior. Pfeiler and Egloff (2018) measured trust, patience, impulsivity, risk aversion, and an optimistic attitude about the future. Food neophobia *via* the “Food Neophobia Scale” (Pliner and Hobden, 1992) and pathogen and moral disgust *via* the “Three-Domain Disgust Scale” (Tybur et al., 2009) were measured by Cliceri et al. (2018). Seeking, play, care, fear, anger, sadness, spirituality, Machiavellianism, non-pathological narcissism, and non-pathological psychopathy were assessed with the “Short Dark Triad Scale” (Jones and Paulhus, 2014) by Sariyska et al. (2019). Hopwood and Bleidorn (2020) investigated diverse antisocial personality features by measuring the “Big Five” characteristics (see above), SDO (see above), negative affectivity, detachment, psychoticism, antagonism, disinhibition, callousness, self-centeredness, entitlement, and indifference. Lastly, daily affect, depressogenic thinking, and self-focused attention are contents of the study of Nezlek et al. (2018), with emotional eating for the study of Cruwys et al. (2020).

#### Values

Allen et al. (2000) used the “Rokeach value survey” (Rokeach, 1973). The importance of respect for status and the avoidance of human suffering was assessed by De Backer and Hudders (2015). Cruwys et al. (2020) examined purity, fairness, authority, care, and loyalty (Davies et al., 2014). Both studies used the moral foundation questionnaire of Graham et al. (2011). Purity, fairness, and authority (in addition to universalism and harm), inspired by the five moral foundations of Graham et al. (2009), were also assessed by Ruby et al. (2013) *via* the “Portrait Value Questionnaire” (Schwartz et al., 2001). Kessler et al. (2018) investigated various values using the “Portraits Value Questionnaire” by Schmidt et al. (2007). Dhar et al. (2008) collected data on ought-to-be and existing values from a cohort of teachers and physicians using the “checklist of values” (Dhar, 1996). Kalof et al. (1999) assessed altruistic and traditional values by means of the “Schwartz value survey” (Schwartz, 1992). The “Schwartz value survey” was also used by Lindeman and Sirelius (2001) to create food choice ideologies. The same study also investigated humanism and normativism using “Tomkins' polarity scale” (De St. Aubin, 1996). Piazza et al. (2015) tested pride and moral self-regard (related to the consumption and use of animal products), and Rothgerber (2015b) assessed ethical idealism through the “ethics position questionnaire” (Forsyth, 1980). The study conducted by Sims (1978) provided information about food-related value orientations, while Pfeiler and Egloff (2018) examined political attitudes. In the study of Trethewey and Jackson (2019), personal health was assessed using a self-designed questionnaire. Daily life satisfaction and the presence of meaning in life were investigated by Nezlek et al. (2018), while current life satisfaction and satisfaction with health were studied by Pfeiler and Egloff (2018). Lastly, Kessler et al. (2018) used the WHO Quality of Life-BREF (World Health Organization, 2014).

#### Empathy

A total of four studies dealt with the topic of empathy. Preylo and Arikawa (2008) and Cliceri et al. (2018) investigated the ability to feel empathy by examining perspective-taking, fantasy, empathetic concern, and personal distress or disease using the “Interpersonal Reactivity Index” (IRI; Davis, 1983). Kessler et al. (2018) applied the short form of the “Empathizing Scale” (Samson and Huber, 2010), and Hopwood and Bleidorn (2020) examined the lack of empathy *via* the “Five Factor Narcissism Inventory” (Glover et al., 2012).

### Outcomes

In the following analysis, only differences between the dietary groups are reported.

#### Personality

All three studies that examined RWA found that omnivorism was associated with significantly greater RWA compared to vegetarianism (Allen et al., 2000; Ruby et al., 2013; Veser et al., 2015). All five studies that measured SDO came to a consistent conclusion that omnivorism was associated with a significantly greater SDO than vegetarianism (Allen et al., 2000; Bilewicz et al., 2011; Piazza et al., 2015; Veser et al., 2015; Hopwood and Bleidorn, 2020). In line with these findings, the study conducted by Veser et al. (2015) indicated a significantly higher tendency to be prejudiced among omnivores than among vegetarians. The study by Hopwood and Bleidorn (2020) suggested that, when compared to vegetarians, omnivores may be more callous, self-centered, entitled, indifferent, less agreeable, and had higher scores in agentic values. Appropriately, Sariyska et al. (2019), in their second sub-study, found that Machiavellianism, non-pathological narcissism, and non-pathological psychopathy (here: no significance after controlling for sex) were significantly higher in omnivores than in vegetarians. In two studies (Rothgerber, 2015a,b), vegetarians were not compared to omnivores but to flexitarians who vary between a vegetarian and omnivorous diet. Interestingly, flexitarians score significantly lower on misanthropy in comparison to vegetarians (Rothgerber, 2015b). Furthermore, flexitarians scored significantly lower on absolutism (in strictly following their diet), violated their diets more often, and felt less ethically associated guilt when doing so than vegetarians (Rothgerber, 2015a). Cruwys et al. (2020) primarily investigated factors relating to the adherence to one's diet and found that vegans who were part of the dietary group least likely to state the facilitator of conscientiousness and a lack of willpower as a barrier to adherence, did not base their diet on an individual context. Furthermore, the most frequently stated that they felt no barriers at all, followed by the vegetarian group. A lack of willpower as a barrier to adherence was mentioned second mostly by the paleo group and most often by weight loss dieters along with mood or emotion. Inconvenience as a barrier was reported most frequently by people on a gluten-free diet. Rosenfeld and Burrow (2018) found vegetarians scoring significantly higher in strictness than omnivores. According to the study conducted by Forestell et al. (2012), vegetarians were significantly more open to new experiences and variety-seeking than omnivores. Pfeiler and Egloff (2018) found indications for vegetarians being significantly more open, higher in trust (here: no significance after controlling for socio-demographic variables), and lower in consciousness in comparison to omnivores. In the study of Forestell and Nezlek (2018), vegetarians also appeared to be significantly more open than semi-vegetarians and omnivores, with semi-vegetarians lying in between. Openness was also examined by Tan et al. (2021), with vegans and vegetarians achieving significantly higher scores than omnivores. In addition, this study found vegans and vegetarians scoring significantly higher on intellect and compassion than omnivores. Vegetarians showed a significantly lower pathogen disgust toward infectious agents and a (not significant) lower moral disgust toward antisocial activities than omnivores and flexitarians in the study conducted by Cliceri et al. (2018). Forestell and Nezlek (2018) found omnivores being significantly less neurotic than vegetarians and semi-vegetarians. Compared to omnivores and semi-vegetarians, vegetarians had lower self-esteem, psychological adjustment, stronger negative moods, more negative social events, and more pronounced thoughts about themselves in the study of Nezlek et al. (2018). Sariyska et al. (2019) revealed that vegetarians had significantly higher scores in care, sadness, and spirituality and lower scores in play (here: no significance after Bonferroni correction). Kessler et al. (2018) found no significant differences in personality between the dietary groups among medical professionals.

#### Values

The outcomes of the reviewed studies were classified by applying the “10 motivational types of values” and by summarizing these values into the following motivational values (Schwartz, 2012):

- Self-transcendence: Universalism, benevolence.
- Conservation: Conformity, tradition, security.
- Self-enhancement: Power, achievement, hedonism.
- Openness to change: Hedonism, stimulation, self-direction.

The findings of the studies were integrated into this model to allow them to be structured according to content. Findings with a *p* < 0.05 were evaluated as significant and are listed below.

*Universalism (Self-transcendence)*. In comparison to omnivorism, vegetarianism was associated with a significantly greater emphasis on universalism (Ruby et al., 2013, among Euro-Americans, Lindeman and Sirelius, 2001), fairness (Ruby et al., 2013, among Indians and Euro-Americans), social justice, equality, and peace (Allen et al., 2000). Compared to flexitarianism, vegetarianism was associated with a significantly greater belief in the importance of the avoidance of human suffering (De Backer and Hudders, 2015). In comparison to veganism, flexitarianism, and ovo-lacto vegetarianism were associated with a significantly lower emphasis on idealism (Rothgerber, 2015b). In comparison to vegetarianism, omnivorism was associated with a significantly greater emphasis on equity (Allen et al., 2000). Compared to vegetarians, omnivores experienced less moral self-regard (Piazza et al., 2015, related to their consumption and use of animal products). In comparison to omnivores, vegetarians expressed a significantly stronger preference for (food-related) ethics, but omnivores expressed a significantly stronger preference for (food-related) economics and the social and psychological uses of food. The “social-psychological uses of food value-orientation was the most negatively (and ethics most positively) associated with vegetarianism” (Sims, 1978; Filippi et al., 2010).

*Benevolence (Self-transcendence)*. Compared to vegetarians, omnivores stated benevolence as an “ought-to-be” value more frequently (Dhar et al., 2008). Vegetarianism was associated with a significantly greater emphasis on love, while omnivorism was associated with a significantly greater emphasis on responsibility (Allen et al., 2000). Altruistic values were significantly more prevalent among vegetarians than among omnivores (Kalof et al., 1999).

*Conformity (Conservation)*. In comparison to vegetarianism, omnivorism was associated with a significantly greater emphasis on self-control (Allen et al., 2000). Compared to omnivores, vegetarians cited discipline as an existing value more frequently (Dhar et al., 2008). Vegetarians advocated the value of avoiding harm significantly more than omnivores (Ruby et al., 2013, among Indians).

*Tradition (Conservation)*. Vegetarianism was significantly negatively correlated with traditional values (Kalof et al., 1999). The study of Pfeiler and Egloff (2018) found vegetarians scoring significantly lower in conservativism and significantly higher in interest in politics than omnivores. In comparison to omnivores, vegetarians adhered significantly stronger to the food-related value orientation of religion (Sims, 1978).

*Security (Conservation*). In comparison to omnivores, vegetarians (and vegans) displayed a significantly stronger preference for (food-related) health (Sims, 1978) and personal-health values (Trethewey and Jackson, 2019). Compared to vegetarians, omnivores had a significantly stronger preference for (food-related) familism (Sims, 1978).

*Power (Self-enhancement)*. Compared to vegetarianism, omnivorism was associated with a greater emphasis on social power (Allen et al., 2000). In comparison to flexitarianism, omnivorism was associated with a greater emphasis on the importance of respect for status (De Backer and Hudders, 2015). Among Indians, vegetarians advocated the value of authority significantly more than omnivores; but among Euro-Americans, vegetarians emphasized the values of authority less than omnivores (Ruby et al., 2013). Compared to omnivores, vegetarians stated wealth as an “ought-to-be” value more frequently (Dhar et al., 2008).

*Achievement (Self-enhancement)*. Omnivores experienced less pride (related to their consumption and use of animal products) compared to vegetarians (Piazza et al., 2015). In comparison to omnivorism, vegetarianism was associated with a greater emphasis on growth and intellectualism and a lower emphasis on logic (Allen et al., 2000). Compared to omnivores, vegetarians held a significantly stronger preference for (food-related) education (Sims, 1978).

*Hedonism (Self-enhancement and openness to change)*. Vegetarianism was associated with a greater emphasis on happiness compared to omnivorism (Allen et al., 2000).

*Stimulation (Openness to change)*. Vegetarianism was associated with a greater emphasis on stimulation (Lindeman and Sirelius, 2001) and excitement compared to omnivorism (Allen et al., 2000).

*Self-Direction (Openness to change)*. In comparison to omnivorism, vegetarianism was associated with a greater emphasis on self-direction (Lindeman and Sirelius, 2001).

The study conducted by Lindeman and Sirelius (2001) grouped different values into food choice ideologies. The findings of the authors demonstrated that the ecological ideology (including universalism, stimulation, and self-direction) was positively associated with vegetarianism and a humanistic view of the world. Cruwys et al. (2020) investigated factors relating to the adherence to one's diet, particularly for vegans who were the dietary group most likely and vegetarians who were second most likely to state the adherence facilitators of ethical or moral concerns and identity. Furthermore, vegetarians least frequently stated health as a facilitator. The paleo group most likely reported enjoyment and second most likely reported health as a facilitator. Health as a barrier was reported most frequently by people on a gluten-free diet. Kessler et al. (2018) found no significant differences in values and/or quality of life between the dietary groups among medical professionals. Lower daily life satisfaction with a marginal significance was reported by vegetarians in comparison to omnivores and semi-vegetarians in Nezlek et al. (2018), but no significant differences were found for current life satisfaction and satisfaction with health in Pfeiler and Egloff (2018).

#### Empathy

The study conducted by Preylo and Arikawa (2008) found that, compared to omnivores, vegetarians scored significantly higher on the subscales for fantasy, personal distress, empathetic concern, and perspective-taking, with the last two being the strongest predictors of vegetarianism. In the study of Cliceri et al. (2018), vegetarians showed significantly higher scores in perspective-taking and (not significantly) in empathic concern, also in comparison to omnivores. Among flexitarians, the values for these two outcomes lay between those of omnivores and vegetarians. De Backer and Hudders (2015) stated that “harm or care focuses on motives to relieve suffering, closely related to empathy” and found that vegetarians put more value on care and empathy compared to flexitarians (Funke, 2003; De Backer and Hudders, 2015). The study of Ruby et al. (2013) found that Indian vegetarians valued the ethic of harm avoidance to a significantly greater degree than Indian omnivores. The indication for omnivores being less empathetic than vegetarians was provided by Hopwood and Bleidorn (2020), while the study by Kessler et al. (2018) found no significant differences in the ability to be empathetic between the dietary groups among medical professionals.

### Risk of Bias in Individual Studies

The risks of bias in the individual studies are presented in Table 2. While 13 studies were given four stars (Kalof et al., 1999; Lindeman and Sirelius, 2001; Preylo and Arikawa, 2008; Forestell et al., 2012; De Backer and Hudders, 2015; Veser et al., 2015; Cliceri et al., 2018; Forestell and Nezlek, 2018; Kessler et al., 2018; Pfeiler and Egloff, 2018; Sariyska et al., 2019; Tan et al., 2021), seven studies were given three stars (Allen et al., 2000; Bilewicz et al., 2011; Rothgerber, 2015a; Nezlek et al., 2018; Cruwys et al., 2020; Tan et al., 2021). Seven studies were given two stars (Sims, 1978; Lindeman and Sirelius, 2001; Ruby et al., 2013; Piazza et al., 2015; Rothgerber, 2015b; Rosenfeld and Burrow, 2018), and four studies were given one star (Dhar et al., 2008; Piazza et al., 2015; Trethewey and Jackson, 2019; Hopwood and Bleidorn, 2020). Sub-studies were considered individually. Since the diet of a participant can only be recorded by means of self-reporting in survey-based research, none of the studies could be given five stars (under the subcategory “is the case definition adequate?”). Notably, most of the studies had significantly larger proportions of female participants. Overall, this is associated with a non-negligible risk of bias. In 7 of the 31 included studies (including sub-studies), there was no control for gender (Dhar et al., 2008; Piazza et al., 2015; Rothgerber, 2015a; Rosenfeld and Burrow, 2018; Trethewey and Jackson, 2019; Hopwood and Bleidorn, 2020). Using the NOS, the remaining 24 studies received one star (^*^) for their consideration of gender distribution in the category “comparability.” Sub-studies were considered individually. According to the quality classes identified in section **Risk of Bias in Individual Studies**, 19 studies were classified as good, 8 studies as fair, and 4 studies as poor quality. In the case of classification intersections, the study in question was assigned to a higher quality level.

**Table 2:** Newcastle-Ottawa scale [76] for case-control studies (^*^ = high quality choice).

**References**	**Selection: adequate definition**		**Selection: representativeness**		**Selection: selection of controls**		Sum of * for selection (max. 3*)		Comparability (max. 2*)		Total of *		**Quality level**
Allen et al. (2000)	Substudy 1:	b	Substudy 1:	a*	Substudy 1:	a*	Substudy 1:	**	Substudy 1:	a*	Substudy 1:	***	Good
	Substudy 2:	b	Substudy 2:	a*	Substudy 2:	a*	Substudy 2:	**	Substudy 2:	a*	Substudy 2:	***	Good
Bilewicz et al. (2011)		b		b		a*		*		a**		***	Fair
Cliceri et al. (2018)		b		a*		a*		**		a**		****	Good
Cruwys et al. (2020)		b		b		a*		*		a**		***	Good
De Backer and Hudders (2015)		b		a*		a*		**		a**		****	Good
Dhar et al. (2008)		b		b		a*		*		b		*	Poor
Forestell et al. (2012)		b		a*		a*		**		a**		****	Good
Forestell and Nezlek (2018)		b		a*		a*		**		a**		****	Good
Hopwood and Bleidorn (2020)	Substudy 2:	b	Substudy 2:	b	Substudy 2:	a*	Substudy 2:	*	Substudy 2:	b	Substudy 2:	*	Poor
Kalof et al. (1999)		b		a*		a*		**		a**		****	Good
Kessler et al. (2018)		b		a*		a*		**		a**		****	Good
Lindeman and Sirelius (2001)	Substudy 1:	b	Substudy 1:	b	Substudy 1:	b	Substudy 1:	-	Substudy 1:	a**	Substudy 1:	**	Fair
	Substudy 2:	b	Substudy 2:	a*	Substudy 2:	a*	Substudy 2:	**	Substudy 2:	a**	Substudy 2:	****	Good
Nezlek et al. (2018)		b		a*		a*		**		a*		***	Good
Pfeiler and Egloff (2018)	Substudy 1:	b	Substudy 1:	a*	Substudy 1:	a*	Substudy 1:	**	Substudy 1:	a**	Substudy 1:	****	Good
Piazza et al. (2015)	Substudy 2:	b	Substudy 2:	a*	Substudy 2:	a*	Substudy 2:	**	Substudy 2:	b	Substudy 2:	**	Fair
	Substudy 4:	b	Substudy 4:	b (MTurk)	Substudy 4:	b (MT)	Substudy 4:	-	Substudy 4:	a*	Substudy 4:	*	Poor
Preylo and Arikawa (2008)		b		a*		a*		**		a**		****	Good
Rosenfeld and Burrow (2018)		b		a*		a*		**		b		**	Fair
Rothgerber (2015a)		b		a*		a*		**		a*		***	Good
Rothgerber (2015b)		b		b (MT)		b (MT)		-		a**		**	Fair
Ruby et al. (2013)	Substudy 1:	b	Substudy 1:	b (MT)	Substudy 1:	b (MT)	Substudy 1:	-	Substudy 1:	a**	Substudy 1:	**	Fair
	Substudy 2:	b	Substudy 2:	b (MT)	Substudy 2:	b	Substudy 2:	-	Substudy 2:	a**	Substudy 2:	**	Fair
Sariyska et al. (2019)	Substudy 1:	b	Substudy 1:	a*	Substudy 1:	a*	Substudy 1:	**	Substudy 1:	a**	Substudy 1:	****	Good
	Substudy 2:	b	Substudy 2:	a*	Substudy 2:	a*	Substudy 2:	**	Substudy 2:	a**	Substudy 2:	****	Good
Sims (1978)		b		b		b		-		a**		**	Fair
Tan et al. (2021)	Substudy 1a:	b	Substudy 1a:	a*	Substudy 1a:	a*	Substudy 1a:	**	Substudy 1a:	a**	Substudy 1a:	****	Good
	Substudy 1b	b	Substudy 1b	b	Substudy 1b	a*	Substudy 1b	*	Substudy 1b	a**	Substudy 1b	***	Good
Trethewey and Jackson (2019)		b		b		a*		*		b		*	Poor
Veser et al. (2015)		b		a*		a*		**		a**		****	Good

## Discussion and Conclusions

### Summary of Evidence

The results of this systematic review indicate that omnivorism is associated with a greater SDO and RWA when compared to vegetarianism. It was found that omnivorism is associated overall with less openness to new experiences and variety-seeking and with a higher tendency to be prejudiced than vegetarianism. Furthermore, it is indicated that the values of vegetarians are based more on hedonism, universalism, stimulation, and self-direction when compared to omnivores, with the last three concepts leading to a stronger ecological ideology, which is positively associated with a humanistic view of the world. Compared to vegetarians, it cannot be ruled out that the values of omnivores are based more on social power (at least among Western countries) as indicated in the study of Allen et al. (2000). Power, as one of the 10 basic values mentioned in section Values, can be described as its central motivational goal in the sense of “social status and prestige, control or dominance over people and resources” (Schwartz, 2012). Matching indications were found by Hopwood and Bleidorn (2020) and Sariyska et al. (2019). In comparison to omnivores, vegetarians tend to be more neurotic, be lower in self-esteem, and have stronger negative moods (Forestell and Nezlek, 2018; Nezlek et al., 2018). There was no clear trend among the dietary groups regarding values based on achievement, security, conformity, benevolence, or tradition. The review showed a further tendency among vegetarians toward higher empathy than those who follow a diet consisting of meat and meat-based products.

### Concordance With Prior Reviews

The findings of this review are partially in line with those of prior reviews that compare dietary habits. In a systematic review by Ruby (2012), diverse findings related to the Western world were presented. Compared to vegetarians, omnivores were found to be more conservative, believed more in traditional values, and revealed a stronger preference for RWA, social hierarchies, and social domination. Compared to omnivores, vegetarians were more liberal and believed more strongly in altruistic values (e.g., environmental protection, equality, and social justice). Vegetarians rejected hierarchical structures, authoritarianism, and violence more frequently and displayed more human-directed empathy than omnivores. In an earlier review, Wilson and Allen (2007) found that vegetarians were less anti-social, more open to new experiences, and ranked emotion as more important than omnivores. Our review also indicates that meat consumption (and, accordingly, omnivorism) might be associated with greater power, inequality, prejudice, hierarchy (e.g., RWA and SDO), and dominance measures and with fewer pro-environmental attitudes. Moreover, Wilson and Allen (2007) present the outcome of a self-conducted study (excluded from this systematic review because of the differentiated consideration of strong, moderate, and weak omnivores) with similar results. Here, strong omnivorism is associated with greater SDO, RWA, and authority measures than vegetarianism.

### External and Internal Validity

The studies included in this review were largely conducted in industrialized Western countries (North America, Europe, Australia, and Oceania), except for two studies (Dhar et al., 2008; Ruby et al., 2013) that were (completely or partially) of Indian origin and, therefore, subject to significant religious influence. For this reason, the results of these two studies are of limited use for the question at hand as religiosity can greatly influence the personality and values of people. Moreover, one of the Indian studies was rated as having poor methodological quality. All the participants of the study were adults, and all the studies included vegetarians.

Twenty-three studies included omnivores, and five studies included flexitarians. All studies obtained information about the diets of participants through self-reporting, which appears to be an adequate means of obtaining such information. Another way of collecting the diets of individuals would only be possible in a limited, supervised, and documented framework (e.g., during an in-patient stay). To the extent that the individual studies made it comprehensible, the classification of the participants into various nutritional groups was consistent with the definitions of different nutritional forms (described in section Background). Studies that used inconsistent definitions were excluded. The transferability of the findings is limited by the moderate-to-high risk of bias in some of the studies. The comparability of the included studies was adequate, with all but five sub-studies (at least partially) meeting the appropriate assessment criteria in this category. Finally, it is noteworthy that 21 of the 31 (sub-) studies met them completely.

## Limitations

Since the topic of vegetarianism on the one hand and personality, on the other hand, involves a broad spectrum of facets, the selected outcomes might only represent a small part of the full picture. There are many interesting, but also very extensive, aspects of the research fields of nutrition, psychology, and psychiatry. Therefore, and to answer a narrowly defined and clear question, we deliberately omitted the topics listed below. The following limitations of this review must be considered, namely, only studies dealing with healthy people were considered and no outcomes that have primarily pathological relevance were included e.g., depression, appearance dissatisfaction, and eating disorders in Lindeman and Stark (1999). Personality was defined as the totality of the personal, characteristic, and individual qualities of a person. Only the individual values of a person were relevant for this work, not social values and norms (e.g., the link between meat and masculinity in Timeo and Suitner, 2017). Additionally, it should be noted again that, in most of the included studies, the proportion of women was higher than that of men. Therefore, the results for collectives with significantly higher numbers of men could be different. Allen et al. (2000) found men having higher scores on the “vegan-omnivore-scale” (i.e., higher consumption of meat). De Backer and Hudders (2015) displayed flexitarian men compared to omnivore men believing more in the importance of respect for status and authority but found reverse findings for women. In addition, Rothgerber (2015b) found females scoring higher on idealism and lower on misanthropy than men. In the study of Forestell and Nezlek (2018), vegetarians and semi-vegetarians showed a greater tendency toward neuroticism than omnivores only among male participants, whereas women were more neurotic than men in general. The relevance of the above-mentioned differences between the dietary groups, taking the generally higher proportion of women in the studies participants and among vegetarians in general (as already shown in many past studies) into account, remains unclear. Consequently, it is possible that the differences in the measured variables can be explained by gender distribution rather than by genuine differences between the dietary groups. Thus, the general widespread socio-psychological differences between genders play an important role in the evaluation of nutritional studies.

Even though the authors are aware that the topics of animal ethics, animal rights, and environmental protection have a clear relation to the question, corresponding study results (e.g., Lund et al., 2016) were deliberately not considered in this review, as these topics are complex and should be discussed elsewhere. Parallel to this, studies that investigated the motivation for a diet (e.g., Kim et al., 1999; De Boer et al., 2017) were excluded. Additionally, the assessable risk of bias was limited, as not all the criteria of the NOS (Wells et al., 2014) could be applied. Particularly, not all of the categories were assessed, especially the category “exposure,” which could not be considered. Therefore, there were fewer maximum achievable stars, and an adjustment of the rating was needed. Of 31 studies (sub-studies included), four studies were poor and eight studies were of fair quality, representing a further limitation of this review. In addition, the pre-existing rating system used to classify the studies into quality grades was exemplified for the evaluation of the NOS for cohort studies and not for case-control studies (used in this review) (Allen et al., 2000). In addition, the search was carried out only *via* the three mentioned databases without carrying out additional steps to reduce the risk of publication bias (e.g., hand search for suitable studies, search for gray literature, or contact with societies or individual authors). Furthermore, the missing pre-registration of the review and the absence of a calculation of the inter-coder reliability within the framework of the various steps in the elaboration of this review could be considered as minor methodological weaknesses of this work. Slight discrepancies among the three authors involved in the phase of study selection were resolved unanimously *via* consensus discussions, but are a potential limitation of this review.

### Implications for Further Research and Conclusions

This systematic review indicates that vegetarians differ from omnivores in their personalities, values, and ability to be empathetic. In view of this, and given the increasing influence of a vegetarian diet on an individual and global level, the subject of vegetarianism merits further research. For example, an interesting aspect could also be to further investigate perceptions of others on vegans, vegetarians, and/or meat reducers to compare the extent to which these are consistent with results such as those found in this review. Patel and Buckland (2021) found that meat reducers were perceived as positive overall by others in terms of their social representations and personality traits, which, in some cases, are more positive than vegetarians and habitual meat-eaters. “These results confirm that […] [meat reducers] are an appropriate referent group for use in future social influence-based interventions aiming to reduce meat intake” (Patel and Buckland, 2021). Also, a critical science-based reflection on our way of eating, especially with regard to its global impact on climate, environment, habitats, and humankind, seems very necessary, with references to the current global crises. For example, factory farming and industrial fishing in the context of a quickly growing global demand for animal products have manifold negative effects on human and planetary health: the emission of climate-damaging greenhouse gases (Intergovernmental Panel on Climate Change, 2014; Food Agriculture Organization of the United Nations, 2017), the high pollution of the environment with pollutants (Umweltbundesamt, 2015; Food Agriculture Organization of the United Nations, 2018), and the deforestation of (rain-) forests for, among other things, the cultivation of animal feed (Fleischatlas, 2021), to name just a few aspects. Thus, sustainable forms of nutrition should receive more consideration as one of the major contributions to solve these global problems (Willett et al., 2019; Benton et al., 2021). According to a few of the findings, flexitarians seem to occupy an intermediary position between omnivores and vegetarians. For example, Forestell et al. (2012) found vegetarians being more open to new experiences and variety-seeking than flexitarians. It is apparent that the numerical values of flexitarians lie between those of vegetarians and omnivores, albeit much closer to the direction of omnivores. Furthermore, there are indications that ovo-lacto vegetarians often occupy an intermediary position between flexitarians and vegans. For example, flexitarians felt less ethical guilt than ovo-lacto vegetarians, while the latter displayed less ethical guilt than vegans (Rothgerber, 2015a). Additionally, flexitarians and ovo-lacto vegetarians scored significantly lower on idealism than vegans, with no differences between flexitarians and ovo-lacto vegetarians (Rothgerber, 2015b). In contrast, the study of Bilewicz et al. (2011) could not find any clear differences between the ovo-lacto vegetarian and the vegan group. Further, RWA and SDO were positively correlated with the “vegan-omnivore scale” used in the study of Allen et al. (2000). This means that greater vegan identification was associated with lower RWA and SDO that, accordingly, increased with greater omnivore identification. Similar results were presented by Veser et al. (2015). Considering these findings, vegans may be a promising field of research as they may produce even more pronounced outcomes on parameters. By considering the findings of the study of Kessler et al. (2016), one reason for this could be the differences in the motivation for adopting a diet. Furthermore, this study found that vegans (compared to ovo-lacto vegetarians) scored lower on neuroticism and higher on openness and empathy. In terms of their values, vegans scored higher on self-determination and universalism and lower on power, achievement, safety, conformity, and tradition (Kessler et al., 2016). It would be interesting to find out if these distributional tendencies can be confirmed in the framework of other new studies.

In addition, differences between genders and their possible influence on the collected target variables should be given even more attention, as they offer great potential for bias. To examine the relationship of measured outcomes with dietary patterns more precisely, it may be useful to consider the duration of the followed diets more often in future projects. For a better assessment of the risks of bias of the individual studies, it would be very useful to develop an appropriate measurement instrument. The NOS is well-established but not yet adapted for survey-based reviews without any exposure factor.

An investigation on alternative eating habits reveals multiple discrepancies regarding the terminology of the dietary groupings. There are different and confusing definitions of the same diet. The terminology of the various nutritional groups is misleading and worthy of reconsideration. For example, the division between flexitarians and semi-vegetarians is an issue that requires further clarification. In the absence of clear definitions, it is likely that a higher number of people define themselves as vegetarians, which could complicate studies on this topic. Another, albeit cost-intensive method of objectifying the diet status is the measurement of biomarkers in, e.g., blood or urine. This provides one option for increasing the validity of future studies. In general, the transparency of the research process of the individual studies and reviews like the present one could be improved by pre-registering them in the future.

## Author's Note

The lead author affirms that this manuscript is an honest, accurate, and transparent account of the study being reported. The reporting of this work is compliant with PRISMA guidelines. The lead author affirms that no important aspects of the study have been omitted and that any discrepancies from the study as planned have been explained.

## Author Contributions

SH drafted the manuscript, performed the data extraction, and editing. CK and HC supported the manuscript draft and the data evaluation. MJ, VM, AM, and DS participated in editing the manuscript. The assessment of the risk of bias was done by SH and DL. All authors revised the manuscript critically and approved the final manuscript.

## Conflict of Interest

The authors declare that the research was conducted in the absence of any commercial or financial relationships that could be construed as a potential conflict of interest.

## Publisher's Note

All claims expressed in this article are solely those of the authors and do not necessarily represent those of their affiliated organizations, or those of the publisher, the editors and the reviewers. Any product that may be evaluated in this article, or claim that may be made by its manufacturer, is not guaranteed or endorsed by the publisher.
